# Prevalence of Malaria and Chikungunya Co-Infection in Febrile Patients: A Systematic Review and Meta-Analysis

**DOI:** 10.3390/tropicalmed6030119

**Published:** 2021-06-30

**Authors:** Wanida Mala, Polrat Wilairatana, Kwuntida Uthaisar Kotepui, Manas Kotepui

**Affiliations:** 1 Medical Technology, School of Allied Health Sciences, Walailak University, Tha Sala, Nakhon Si Thammarat 80160, Thailand; wanida.ma@wu.ac.th (W.M.); kwuntida.ut@wu.ac.th (K.U.K.); 2 Department of Clinical Tropical Medicine, Faculty of Tropical Medicine, Mahidol University, Bangkok 73170, Thailand; polrat.wil@mahidol.ac.th

**Keywords:** malaria, *Plasmodium*, chikungunya, CHIKV, co-infection

## Abstract

**Background**: Co-infection with malaria and chikungunya could exert a significant public health impact with infection misdiagnosis. Therefore, this study aimed to collect qualitative and quantitative evidence of malaria and chikungunya co-infection among febrile patients. **Methods:** Potentially relevant studies were identified using PubMed, Web of Science, and Scopus. The bias risk of the included studies was assessed using the checklist for analytical cross-sectional studies developed by the Joanna Briggs Institute. The pooled prevalence of malaria and chikungunya co-infection among febrile patients and the pooled prevalence of chikungunya virus (CHIKV) infection among malaria patients were estimated with the random effect model. The odds of malaria and chikungunya co-infection among febrile patients were also estimated using a random effect model that presumed the heterogeneity of the outcomes of the included studies. The heterogeneity among the included studies was assessed using the Cochran Q test and I^2^ statistics. Publication bias was assessed using the funnel plot and Egger’s test. **Results:** Of the 1924 studies that were identified from the three databases, 10 fulfilled the eligibility criteria and were included in our study. The pooled prevalence of malaria and chikungunya co-infection (182 cases) among febrile patients (16,787 cases), stratified by diagnostic tests for CHIKV infection, was 10% (95% confidence interval (CI): 8–11%, I^2^: 99.5%) using RDT (IgM), 7% (95% CI: 4–10%) using the plaque reduction neutralization test (PRNT), 1% (95% CI: 0–2%, I^2^: 41.5%) using IgM and IgG ELISA, and 4% (95% CI: 2–6%) using real-time RT-PCR. When the prevalence was stratified by country, the prevalence of co-infection was 7% (95% CI: 5–10%, I^2^: 99.5%) in Nigeria, 1% (95% CI: 0–2%, I^2^: 99.5%) in Tanzania, 10% (95% CI: 8–11%) in Sierra Leone, 1% (95% CI: 0–4%) in Mozambique, and 4% (95% CI: 2–6%) in Kenya. The pooled prevalence of CHIKV infection (182 cases) among malaria patients (8317 cases), stratified by diagnostic tests for CHIKV infection, was 39% (95% CI: 34–44%, I^2^: 99.7%) using RDT (IgM), 43% (95% CI: 30–57%) using PRNT, 5% (95% CI: 3–7%, I^2^: 5.18%) using IgM and IgG ELISA, and 9% (95% CI: 6–15%) using real-time RT-PCR. The meta-analysis showed that malaria and chikungunya co-infection occurred by chance (*p*: 0.59, OR: 0.32, 95% CI: 0.6–1.07, I^2^: 78.5%). **Conclusions:** The prevalence of malaria and chikungunya co-infection varied from 0% to 10% as per the diagnostic test for CHIKV infection or the country where the co-infection was reported. Hence, the clinicians who diagnose patients with malaria infections in areas where two diseases are endemic should further investigate for chikungunya co-infection to prevent misdiagnosis or delayed treatment of concurrent infection.

## 1. Introduction

Chikungunya is one of the most common vector-borne infectious diseases caused by the chikungunya virus (CHIKV) [1,2]. The clinical symptoms of CHIKV infection are similar to those of other acute febrile illnesses (AFIs), such as dengue, leptospirosis, scrub typhus infection, Zika virus disease, and malaria. These overlapped symptoms include fever, rash, severe joint pain (arthralgia), headache, nausea, and fatigue muscle pain [1,3,4]. Hence, there is a high possibility of misdiagnosis in the case of these AFIs, wherein dual infections are misdiagnosed as a mono-infection, especially in the tropical and subtropical regions where all these AFIs occur [5,6]. The most recent systematic review has identified cases of malaria, dengue, and chikungunya co-infections; it also showed that malaria and dengue co-infection was the most common co-infection, followed by the dengue/chikungunya, malaria/chikungunya, and malaria/dengue/chikungunya co-infections [2]. Importantly, previous studies have shown that the prevalence of a co-infection of AFIs, such as the co-infection of malaria and dengue, could lead to severe diseases [7,8]. However, the impact of malaria and chikungunya is poorly understood. Co-infection with malaria and chikungunya could have significant public health impacts if the infection is misdiagnosed [2]. The misdiagnosis of this co-infection could lead to a delay in either diagnosis or treatment and may potentially result in fatal outcomes. To our knowledge, there is a lack of sufficient evidence on how co-infection affects disease severity and outcomes. Moreover, a systematic review with a meta-analysis on malaria and chikungunya has not yet been conducted. Therefore, in the present study, we aimed to collect qualitative and quantitative evidence of malaria and chikungunya co-infection among febrile patients. Furthermore, the pooled prevalence of chikungunya infection among patients with malaria was also investigated. Finally, the odds of malaria and chikungunya co-infection were determined in order to understand whether the co-infection occurred by chance or by probability.

## 2. Methods

### 2.1. Protocol and Registration

This systematic review and meta-analysis were performed as per the preferred reporting items for systematic reviews and meta-analyses (PRISMA) guidelines [9]. This systematic review was registered at PROSPERO with ID CRD42021258303.

### 2.2. Information Sources and Search Strategy

Potentially relevant studies were identified through PubMed, Web of Science, and Scopus without any restrictions by language or publication date. The combination of the search terms with Boolean operators “(Malaria OR plasmodium) AND Chikungunya” was used to search for the studies. The Medical Subject Heading database was used to check the search terms for accuracy as well as to search for further studies to be included in the present analysis. The search terms and search strategy are shown in Supplementary Material Table S1. To assure that all the relevant studies in the literature were reviewed, we further performed a search in Google Scholar and reviewed the reference lists of the enrolled studies.

### 2.3. Eligibility Criteria

Studies were included as per the inclusion and exclusion criteria. The inclusion criteria were based on the participants (P), outcome of interest (I), and contexts (Co) (PICo): P represented febrile participants or malaria patients; I represented malaria; chikungunya represented co-infection or CHIKV infection; Co represented the global prevalence. All types of study designs, including observational, cohort, and case-control designs, that reported the co-infection of both pathogens were considered. Case reports, case series, letters to editors, comments, reviews, systematic reviews, meta-analyses, studies without full text, and studies from which the data could not be extracted were excluded.

### 2.4. Study Selection

Following the identification of potentially relevant studies from the databases, the duplicates were removed, and the remaining studies were screened for titles and abstracts. The unrelated studies were excluded, and the remaining studies were examined for their full texts. The studies that did not fulfill the eligibility criteria were excluded, with the reasons noted. The studies that fulfilled the eligibility criteria were included for further data extraction and quality assessment. Studies were selected independently by two authors (MK and WM). Any disagreement between the two reviewers was resolved by the third author (PW).

## 3. Data Extraction

Data regarding the following aspects were extracted from the included studies: the name of the first author, the year of publication, the site of the study, the year of the study, the design of the study, the number and characteristics of participants, the age of the subjects, the sex of the subjects, the number of co-infections, the number of malaria mono-infections, the number of CHIKV infections, and the diagnostics tests for malaria and CHIKV infections. Data were extracted by two authors (MK and WM) and were cross-checked by the third author (PW). The extracted data were recorded in the pilot standardized datasheet for further analyses.

### 3.1. Risk of Bias

The bias risk in the included studies was assessed using the checklist for analytical cross-sectional studies that was developed by the Joanna Briggs Institute [10]. The risk of bias for each study was assessed using the following 8 checklist points: (1) the inclusion criteria for sample selection was clearly defined; (2) the details of the study subjects and the setting were provided; (3) the exposure was measured validly and reliably; (4) objective, standard criteria were used for the measurement of the condition; (5) confounding factors were identified; (6) strategies used to deal with confounding factors were described; (7) the outcomes were measured validly and reliably; and (8) appropriate statistical analyses were used. A score of 7–8 was assigned to studies that fulfilled at least 7 checklist item, indicating a low bias risk; studies that fulfilled 5–6 checklist items had a moderate bias risk, and those that fulfilled <5 checklist items had a high bias risk and were excluded from the present study.

### 3.2. Outcomes

The primary outcome of the present study was the pooled prevalence of malaria and chikungunya co-infection in febrile patients. The secondary outcome was the pooled prevalence of CHIKV infection in malaria patients. The third outcome was the odds of malaria and chikungunya co-infection in febrile patients.

### 3.3. Data Synthesis

The qualitative and quantitative collection of evidence from the literature has been described elsewhere [11,12,13]. The quantitative analysis of the evidence was performed for the pooled prevalence of malaria and chikungunya co-infection in febrile patients, and the pooled prevalence of CHIKV infection in malaria patients was estimated using the random effect model (DerSimonian and Laird). The odds of malaria and chikungunya co-infection in febrile patients were estimated using the random effect model for the number of patients with co-infection, malaria without CHIKV infection, CHIKV infection without malaria infection, and febrile patients without malaria or CHIKV infection. The point estimate (pooled prevalence or odds ratio) and the 95% confidence intervals (CIs) for each study have been shown using a forest plot. The heterogeneity in the included studies was assessed using the Cochran Q test and I^2^ statistics. A Cochran Q test with *p* < 0.1 or an I^2^ statistic >25% indicated substantial heterogeneity in the outcome of the included studies. The subgroup analyses of countries and diagnostic tests for CHIKV infection were performed to explore the source of the outcome heterogeneity. All the analyses were performed using Stata version 14 (StataCorp, College Station, TX, USA).

## 4. Results

### 4.1. Search Results

**Figure 1:**
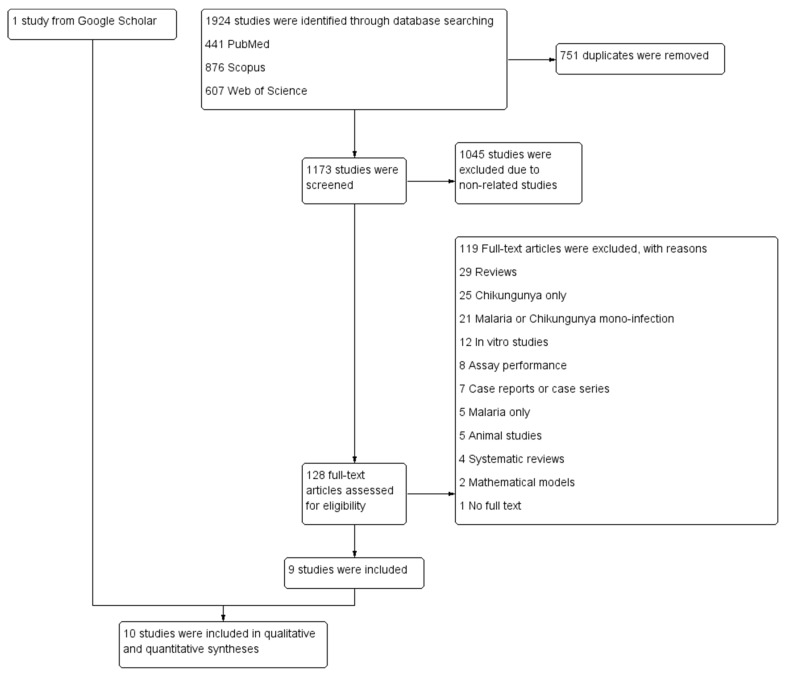
Study flow diagram.

A total of 1924 studies were identified from the following 3 databases: 441 from PubMed, 876 from Scopus, and 607 from Web of Science. After the duplicates were removed, 1173 studies were screened through the titles and abstracts; thereafter, 1045 studies were excluded because they were irrelevant. The remaining 128 studies were examined through their full texts. Of these, 119 studies were excluded because they were reviews (29), only reported on chikungunya (25), only reported on malaria or chikungunya mono-infection (21), were in vitro studies (12), performed assays (8), were case reports or case series (7), reported on malaria only (5), were animal studies (5), were systematic reviews (4), involved mathematical models (2), or did not have full text (1). Thus, 9 studies [14,15,16,17,18,19,20,21,22] that met the eligibility criteria were included. Another study [23], identified from Google Scholar, was included. Finally, 10 studies [14,15,16,17,18,19,20,21,22,23] were included for the qualitative and quantitative analyses (Figure 1).

### 4.2. Characteristics of the Included Studies

All the characteristics of the included studies are shown in Table 1. The studies were published between 2013 and 2021. Most of the included studies [14,15,16,17,18,19,20,21,22] were performed in Africa (9/10, 90%), while only 1 was conducted in Venezuela, in 2018 [23]. Among studies conducted in Africa, 2 were performed in Nigeria in 2008 and 2014 [14,15], 1 in Sudan in 2018 [16], 2 in Tanzania in 2013 and 2015 [17,19], 1 in Sierra Leone from 2012–2013 [18], 1 in Mozambique in 2016 [20], 1 in Senegal from 2009–2013 [21], and 1 in Kenya from 2014–2015) [22]. Most of the included studies were cross-sectional studies (8/10, 80%) [14,15,17,18,19,20,22,23], while the remaining were prospective observational studies [16,21]. Most of the included studies enrolled febrile patients (8/10, 80%), while the remaining enrolled patients suspected to have chikungunya-like illness [16] and those who had malaria [23]. Seven studies enrolled patients across all age ranges (7/10, 70%), while the remaining enrolled only pediatric subjects [17,22] or only adult subjects [23]. For malaria diagnosis, 7 studies [14,15,17,19,21,22,23] used microscopy only or in combination with other methods, while 3 studies [16,18,20] used RDT only. For CHIKV infection diagnosis, 4 studies used IgM and IgG ELISA [7,19,20,23]; 2 used RDT (IgM) [14,18], reverse transcriptase polymerase chain reaction (RT-PCR) [16,22], and plaque reduction neutralization test (PRNT) [15]; and 1 used both IgM ELISA and real-time RT-PCR [21].

### 4.3. Risk of Bias

The risk of bias among the included studies was assessed using the checklist for analytical cross-sectional studies that was developed by the Joanna Briggs Institute (11). Most of the included studies had a moderate bias risk level (15–21, 24), while 2 (22, 23) had low bias risk levels (Table S2, Table 1).

### 4.4. Prevalence of Malaria and Chikungunya Co-Infection among Febrile Patients

**Figure 2:**
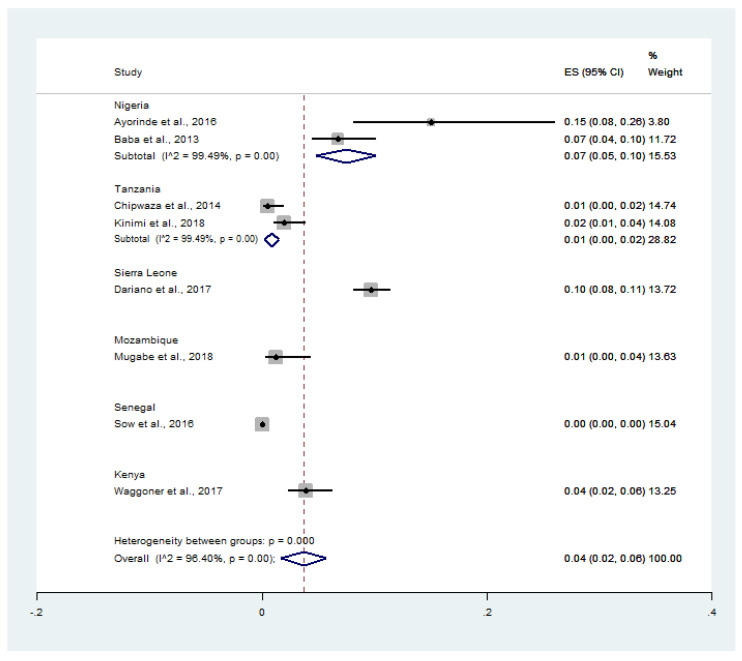
Prevalence of malaria and chikungunya co-infection among febrile patients by countries: % Weighted, the impact proportion of each study to the pooled effect; black dot symbol on the black horizontal line, the point estimate for each study; the black horizontal line, CI; the white diamond symbol, pooled prevalence; CI, confidence interval; ES, effect size (prevalence).

The pooled prevalence of malaria and chikungunya co-infection (182 cases) in febrile patients (16,787 cases) was estimated using 8 studies (15, 16, 18–23). The results showed that the prevalence of co-infection stratified by country was 7% (95% CI: 5–10%, I^2^: 99.5%) in Nigeria, 1% (95% CI: 0–2%, I^2^: 99.5%) in Tanzania, 10% (95% CI: 8–11%) in Sierra Leone, 1% (95% CI: 0–4%) in Mozambique, and 4% (95% CI: 2–6%) in Kenya (Figure 2).

**Figure 3:**
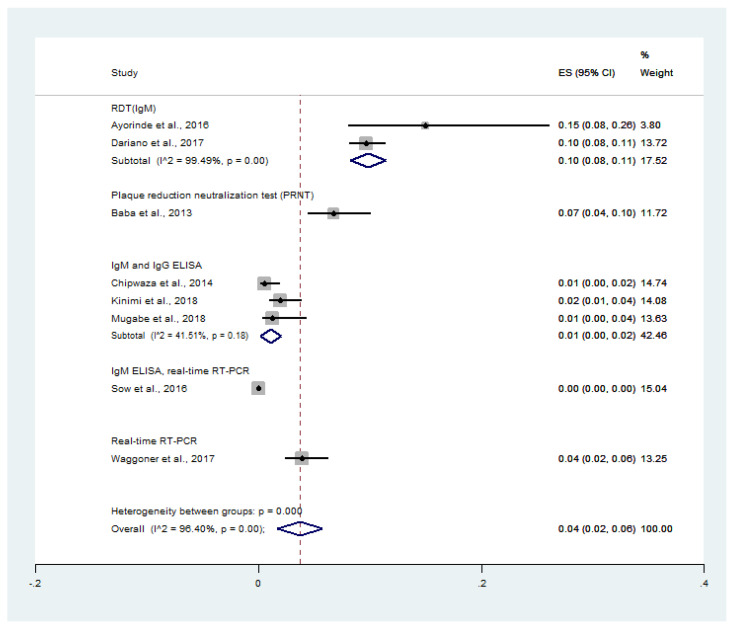
Prevalence of malaria and chikungunya co-infection among febrile patients using diagnostic tests: % weighted, the impact proportion of each study to the pooled effect; the black dot symbol on the black horizontal line, the point estimate for each study; the black horizontal line, CI; the white diamond symbol, the pooled prevalence; CI, confidence interval; ES, effect size (prevalence).

When the prevalence was stratified as per the diagnostic tests for CHIKV infection, the prevalence of co-infection was 10% (95% CI: 8–11%, I^2^: 99.5%) using RDT (IgM), 7% (95% CI: 4–10%) using PRNT, 1% (95% CI: 0–2%, I^2^: 41.5%) using IgM and IgG ELISA, and 4% (95% CI: 2–6%) using real-time RT-PCR (Figure 3).

**Figure 4:**
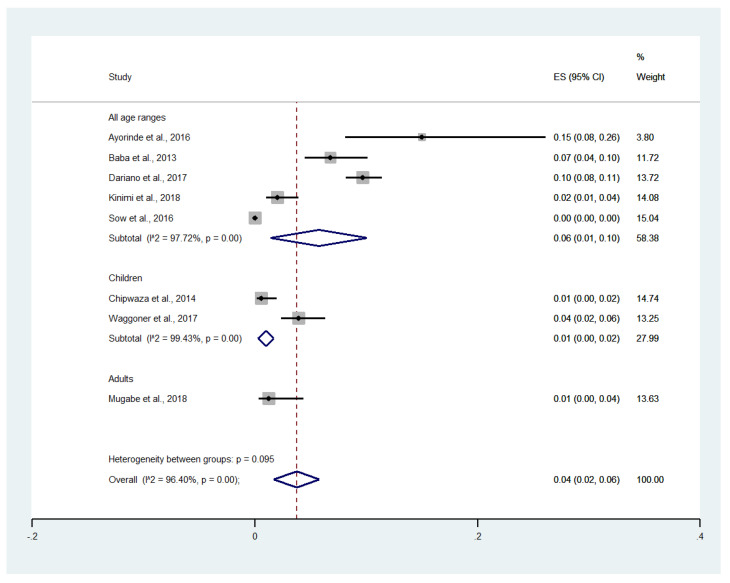
Prevalence of malaria and chikungunya co-infection among febrile patients by age group: % weighted, the impact proportion of each study to the pooled effect; the black dot symbol on the black horizontal line, the point estimate for each study; the black horizontal line, CI; the white diamond symbol, the pooled prevalence; CI, confidence interval; ES, effect size (prevalence).

When the prevalence was stratified as per age groups, the prevalence of malaria and chikungunya co-infection was 6% (CI: 1–10%, I^2^: 99.7%) in studies that enrolled patients of all age groups, 1% (CI: 0–2%, I^2^: 99.4%) in studies that enrolled only pediatric subjects, and 1% (CI: 0–4%) in the study that enrolled only adult subjects (Figure 4). Overall, the pooled prevalence of malaria and chikungunya co-infection in febrile patients was 4% (95% CI: 2–6%, I^2^: 96.4%).

### 4.5. Prevalence of CHIKV Infection among Malaria Patients

**Figure 5:**
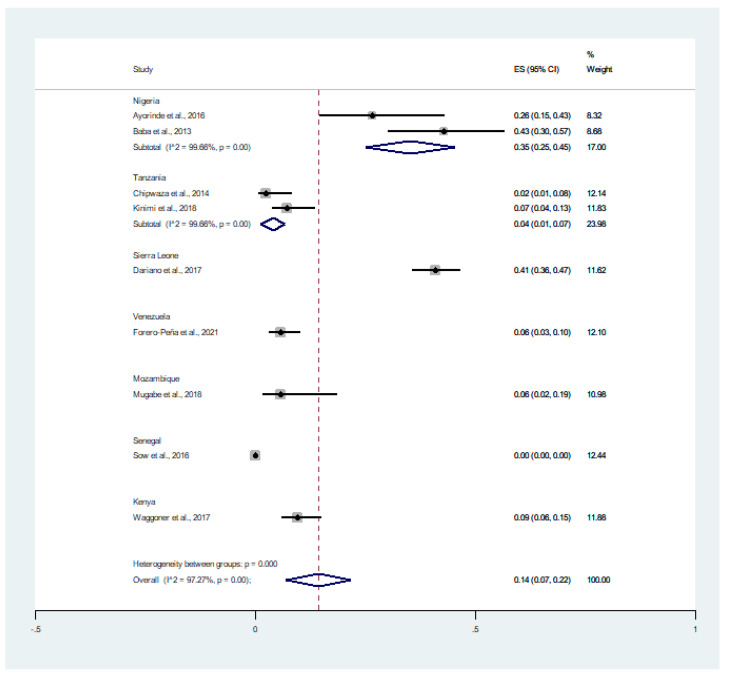
Prevalence of CHIKV infection among malaria patients by country: % weighted, the impact proportion of each study to the pooled effect; the black dot symbol on the black horizontal line, the point estimate for each study; the black horizontal line, CI; the white diamond symbol, the pooled prevalence; CI, confidence interval; ES, effect size (prevalence).

The pooled prevalence of CHIKV infection (182 cases) among malaria patients (8317 cases) was estimated using 9 studies [14,15,17,18,19,20,21,22,23]. The prevalence of CHIKV infection in malaria-positive patients, stratified by country, was 35% (95% CI: 25–45%, I^2^: 99.7%) in Nigeria, 4% (95% CI: 1–7%, I^2^: 99.7%) in Tanzania, 41% (95% CI: 36–47%) in Sierra Leone, 6% (95% CI: 3–10%) in Venezuela, 6% (95% CI: 2–19%) in Mozambique, and 9% (95% CI: 6–15%) in Kenya (Figure 5).

**Figure 6:**
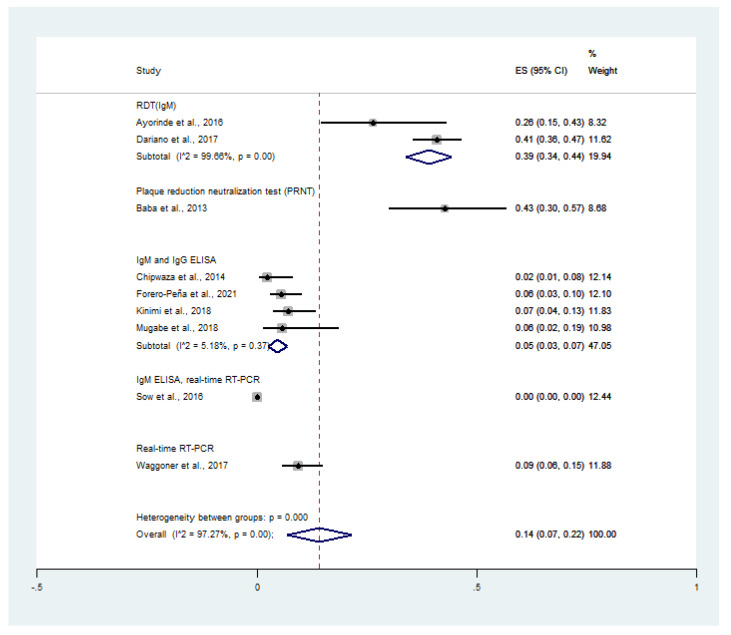
Prevalence of CHIKV infection among malaria patients by diagnostic tests: % weighted, the impact proportion of each study to the pooled effect; the black dot symbol on the black horizontal line, the point estimate for each study; the black horizontal line, CI; the white diamond symbol, the pooled prevalence; CI, confidence interval; ES, effect size (prevalence).

When the prevalence was stratified as per diagnostic tests for CHIKV infection, the prevalence of CHIKV infection in malaria patients was 39% (95% CI: 34–44%, I^2^: 99.7%) using RDT (IgM), 43% (95% CI: 30–57%) using PRNT, 5% (95% CI: 3–7%, I^2^: 5.18%) using IgM and IgG ELISA, and 9% (95% CI: 6–15%) using real-time RT-PCR (Figure 6).

**Figure 7:**
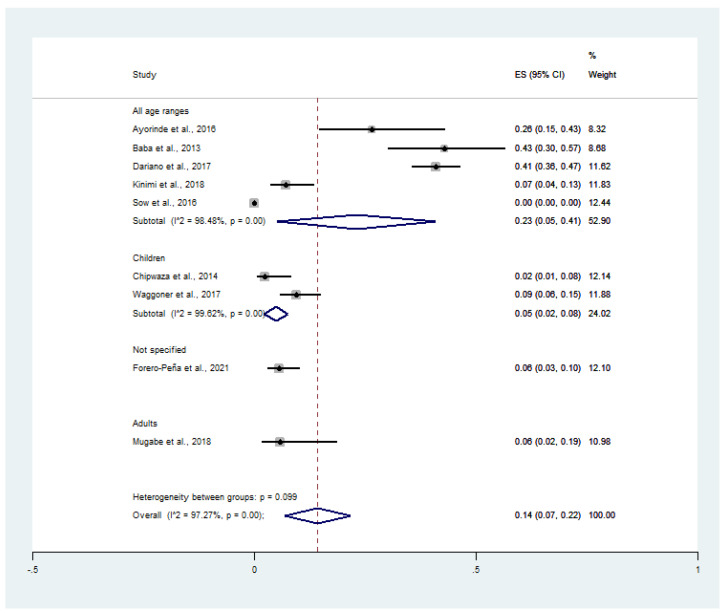
Prevalence of CHIKV infection among malaria patients by age groups: % weighted, the impact proportion of each study to the pooled effect; the black dot symbol on the black horizontal line, the point estimate for each study; the black horizontal line, CI; the white diamond symbol, the pooled prevalence; CI, confidence interval; ES, effect size (prevalence).

When the prevalence of CHIKV infection in malaria patients was stratified by age group, the prevalence of CHIKV infection in malaria patients was 23% (95% CI: 5–41%, I^2^: 98.5%) in studies that recruited patients in all age groups, 5% (95% CI: 2–8%) in children, 6% (95% CI: 2–19%) in adults, and 6% (95% CI: 3–10%) in the study that did not specify the patient age (Figure 7) Overall, the pooled prevalence of CHIKV infection in malaria patients was 14% (95% CI: 7–22%, I^2^: 97.3%).

### 4.6. Odds of Malaria and Chikungunya Co-Infections

**Figure 8:**
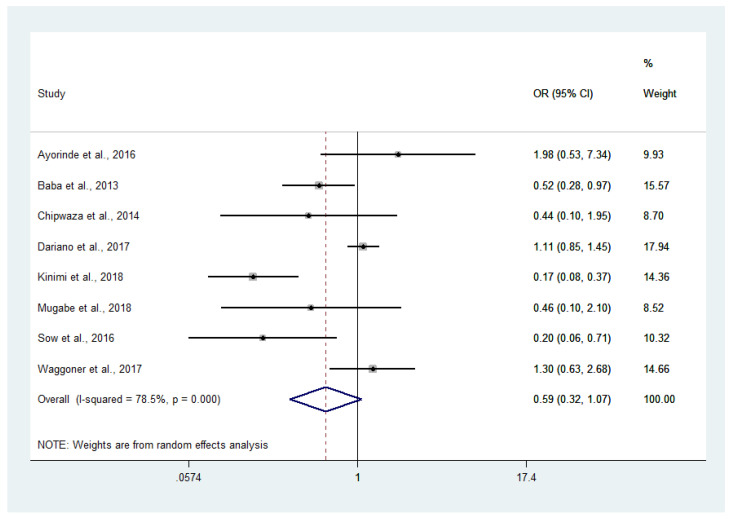
Odds of malaria and chikungunya co-infections: % weighted, the impact proportion of each study to the pooled effect; the black dot symbol on the black horizontal line, the point estimate for each study; the black horizontal line, CI; the white diamond symbol, the pooled prevalence; CI, confidence interval; ES, effect size (prevalence).

The odds of malaria and chikungunya co-infection (182 cases) in febrile patients (16,787 cases) were estimated using 8 studies [14,15,17,18,19,20,21,22]. There were lower odds of co-infection in the studies conducted in Nigeria (OR: 0.52, 95% CI: 0.28–0.97) [15], Tanzania (OR: 0.17, 95% CI: 0.08–0.37) [19], and Senegal (OR: 0.20, 95% CI: 0.06–0.71) [21], while there was no difference in the odds of co-infection in the remaining studies. Overall, the meta-analysis showed that malaria and chikungunya co-infection occurred by chance (*p*: 0.59, OR: 0.32, 95% CI: 0.6–1.07, I^2^: 78.5%) (Figure 8).

### 4.7. Meta-Regression

Meta-regressions with sex as a covariate for the effect estimates were performed. The meta-regressions with sex as a covariate for the pooled prevalence of malaria and chikungunya co-infection in febrile patients showed that sex did not confound the effect estimate (*p*: 0.954, coefficient <0.001, standard error: 0.002). The meta-regressions with sex as a covariate for the pooled prevalence of chikungunya co-infection in patients with malaria showed that sex did not confound the effect estimate (*p*: 0.821, coefficient: 0.001, standard error: 0.006).

### 4.8. Publication Bias

Publication bias in the studies could not be assessed because the number of included studies for the outcome was <10 [24].

## 5. Discussion

In this study, we investigated the prevalence of malaria and chikungunya co-infection in febrile patients from 2013–2021. The results revealed a low (4%) overall pooled prevalence of malaria and leptospirosis co-infection. The subgroup of countries showed that the prevalence ranged from 1% to 10% in several countries within Africa, including Sierra Leone, Nigeria, Kenya, Tanzania, and Mozambique. The results were consistent with those that previously reported a prevalence of 0.02–15% for the co-infection of malaria and chikungunya in Africa [2]. The highest prevalence was demonstrated in Sierra Leone (10%), followed Nigeria (7%), when compared to the prevalence rates in other countries. The pooled prevalence of CHIKV infection in malaria patients was high (14%). The prevalence was 41% in Sierra Leone, followed by 35% in Nigeria in comparison to those in other countries, as follows: 9% in Kenya, 6% in Mozambique, 6% in Venezuela, and 4% in Tanzania. Based on these results, CHIKV infection in patients with febrile illness was common in Africa and America. There have been reports of the spread of CHIKV infection in areas where malaria is endemic, which increases the prevalence of malaria and chikungunya co-infection [25]. These populations include tropical areas in Africa, Latin America, India, and Southeast Asia [25]. In Sierra Leone, the highest occurrence of malaria and CHIKV co-infection was shown by the presence of co-infection in 118 patients from among 1260 febrile patients [2]. Moreover, the co-infections of malaria, chikungunya, and dengue were reported in Sierra Leone, Nigeria, and India [2]. African cohort studies showed that most malaria and chikungunya co-infection seems to be estimated only when the diagnosis for malaria infection is the negative result [25]. In addition, travelers returning from endemic areas as well as immigrants increase the spread of the disease [1,2]. Previous studies showed that age and sex contributed to disease severity [26,27]. The subgroup analysis that included age groups showed a high prevalence of malaria and chikungunya co-infection in febrile patients and a higher prevalence of CHIKV infection in patients with malaria in studies that recruited patients of all age groups, as compared to those in studies that recruited only children or adults. These results indicate that the co-infection of these two diseases could occur in all age groups. However, two studies [19,21] that recruited patients of all age groups reported a low prevalence of co-infection. Therefore, patients of all age groups can be infected by these two pathogens, and age is not the source of heterogeneity in the prevalence of co-infection in febrile patients. In addition, the meta-regressions that included sex showed that sex did not confound the effect estimate. This result indicated that patients of both sexes could be infected with this concurrent infection.

Malaria and chikungunya co-infection are rare; however, single infections share similar clinical symptoms with other febrile illnesses, which leads to misdiagnosis at the early stages of infection [1,2]. In Ethiopia, febrile patients are often misdiagnosed with malaria or typhoid fever [1]. In this study, the prevalence of co-infection was stratified by diagnostic tests for CHIKV infections, including RDT (IgM), PRNT, ELISA (IgM and IgG), and RT-PCR. The highest prevalence of co-infection and CHIKV infection among malaria patients was diagnosed using RDT and PRNT. The lowest prevalence of co-infection and CHIKV infection in malaria patients was diagnosed using ELISA. Currently, CHIKV infection is identified using viral genome detection, viral cultures, and serological tests [28]. RT-PCR is the most common method for diagnosis at the early phase of CHIKV infection. This method can detect the viral RNA of CHIKV within five to seven days after infection, which represents the viremic phase of the disease [28]. The limitation of RT-PCR is that there is low viremia in the serum that is collected more than seven days after the onset of the illness [28]. The RDT and ELISA are used to detect IgM and/or IgM that are more accurate methods of diagnosis after the acute phase of the disease [28,29]. These tests could have low sensitivity when the detection of infection occurs in patients within the acute phase [30]. In addition, ELISA might have the limitations of cross-reactivity of antibodies compared to the other viral infections, including the dengue virus, in particular [29,31]. Therefore, RT-PCR may have high sensitivity for the detection of acute CHIKV infection compared to the serological test, ELISA [28]. However, the prevalence of CHIKV infection may be underestimated due to the capacity of tests and limited facilities in the laboratory [32]. Using RT-PCR is suitable for assessing the serum collected during the first week after the infection as compared to the serological test [29]. In addition, the viral RNA detection test may show negative results because of the time that passes between serum collection and illness onset [28]. This might be confirmed by the detection of the presence of IgG antibodies in patients [28,29]. PRNT is used to measure the titer of neutralizing antibodies in the serum by counting the number of plaques of viral infection [33]. This test has been used conventionally in laboratories for vaccine efficacy that requires the hazardous live CHIKV, and requires fewer days to complete when compared to the RDT [33,34]. Currently, neutralization assays, such as the non-infectious virus replicon particles and pseudotyped viruses, have been developed [35,36]. CHIKV infection shares clinical symptoms with malaria and other AFIs; this may lead to the misdiagnosis of CHIKV infection as malaria and thus lead to increased mortality and morbidity in patients [1,2]. Therefore, the accurate diagnosis of CHIKV infection at the early stage of the infection is crucial in endemic areas.

The meta-analysis showed that malaria and chikungunya co-infection occurred by chance. This could be explained by the fact that these two diseases were transmitted through two different vectors. Malaria is transmitted by the female *Anopheles* mosquitoes, while chikungunya is transmitted via the bite of *Aedes* mosquitoes. However, lower odds of co-infection were demonstrated in the studies conducted in Nigeria [15], Tanzania [19], and Senegal [28]. The results of these studies indicated that there was a very low probability of malaria and chikungunya co-infection, or that one infection might suppress another infection. In endemic areas, where the vectors of malaria and chikungunya co-exist, a high number of asymptomatic malaria and CHIKV infections could aggravate concurrent infection [7,8]. In addition, more severe complications might occur, as shown in a previous study on malaria and dengue co-infection [8].

The present study has certain limitations. First, a limited number of studies reported malaria and chikungunya co-infection; thus, only a single outcome of the meta-analysis could be estimated: the pooled prevalence of the co-infection. Second, some studies might have been missed during study selection. However, we performed searches on other sources, such as Google Scholar, and reviewed the reference lists of the included studies to prevent overlooking relevant studies. Third, differences based on clinical data, laboratory alterations, or treatment outcomes could not be assessed due to the unavailability of data; therefore, future prospective studies should examine these differences to provide relevant data to clinicians that can enable treatment decisions for patients with co-infection.

## 6. Conclusions

The prevalence of malaria and chikungunya co-infection ranged from 0% to 10%, as per diagnostic tests for CHIKV infection or the country where the co-infection was reported. Hence, clinicians who diagnose patients with malaria in areas that are endemic for these two infections should further investigate for chikungunya co-infection to prevent misdiagnosis or delayed treatment of concurrent infection.

## Figures and Tables

**Table 1:** Characteristics of the included studies.

Author	Study Site	Year of Conducted	Study Design	Participants	Age	% Male	Co-Infection	All Malaria Cases	Malaria without CHIKV Infection	CHIKV Infection without Malaria	Test for Malaria	Test for CHIKV	Risk of Bias (High, Moderate, Low)
Ayorinde et al., 2016	Nigeria	2014	Cross-sectional study	60 febrile patients	3–70 years	26.7	9	34	25	4	Microscopy, RDT, PCR	RDT (IgM)	Moderate
Baba et al., 2013	Nigeria	2008	Cross-sectional study	310 febrile patients	<1–80 years	45.2	21	49	28	154	Microscopy	Plaque reduction neutralization test (PRNT)	Moderate
Bower et al., 2021	Sudan	2018	Prospective observational study	102 adults and 40 children presenting with chikungunya-like illness	4 months–70 years		34	39	5	84	RDT	Real-time RT-PCR	Moderate
Chipwaza et al., 2014	Tanzania	2013	Cross-sectional study	364 febrile patients	2–13 years	51.1	2	83	81	15	Microscopy	IgM and IgG ELISA	Moderate
Dariano et al., 2017	Sierra Leone	2012–2013	Cross-sectional study	1260 febrile patients	6–45 years	NS	122	298	176	370	RDT	RDT (IgM)	Moderate
Forero-Peña et al., 2021	Venezuela	2018	Cross-sectional study	161 malaria-positive cases	Mean 34 years	NS	9	161	152	NS	Microscopy	IgM and IgG ELISA	Moderate
Kinimi et al., 2018	Tanzania	2015	Cross-sectional study	400 febrile patients	1–50 years	38	8	112	104	89	Microscopy, RDT	IgM and IgG ELISA	Moderate
Mugabe et al., 2018	Mozambique	2016	Cross-sectional study	163 febrile patients	≥5 years	39.3	2	35	33	15	RDT	IgM and IgG ELISA	Moderate
Sow et al., 2016	Senegal	2009–2013	Prospective observational study	13,845 febrile patients (7387 malaria and 44 arboviral-infected individuals)	1–90 years	20	3	7387	7384	13	Microscopy, RDT	IgM ELISA, real-time RT-PCR	Low
Waggoner et al., 2017	Kenya	2014–2015	Cross-sectional study	385 febrile patients	<18 years	49.9	15	158	143	17	Microscopy, PCR	Real-time RT-PCR	Low

## Data Availability

All data relating to the study in this manuscript are available.

## Supplementary Materials

The following are available online at https://www.mdpi.com/article/10.3390/tropicalmed6030119/s1, Table S1: Search term, Table S2: Quality of the included studies, Checklist S1: PRISMA Checklist.
