# Supplementary material for: Prevalence of Malaria and Chikungunya Co-Infection in Febrile Patients: A Systematic Review and Meta-Analysis
Source: Trop Med Infect Dis. 2021 Jun 30;6(3):119. doi: 10.3390/tropicalmed6030119 (PMC8293423; doi:10.3390/tropicalmed6030119)
Supplement: Supplementary file 1 [file tropicalmed-06-00119-s001.zip › Table S1. Search term.pdf]

Prevalence of malaria and Chikungunya co-infection among febrile patients: a systematic review and meta-analysis

Wanida Mala[1], Polrat Wilairatana[2], Kwuntida Uthaisar Kotepui[1], Manas Kotepui[1*]

[1]Medical Technology, School of Allied Health Sciences, Walailak University, Tha Sala, Nakhon Si Thammarat, Thailand

[2]Department of Clinical Tropical Medicine, Faculty of Tropical Medicine, Mahidol University, Bangkok, Thailand

[*]**Corresponding author**

Manas Kotepui; manas.ko@wu.ac.th, Tel.: +66954392469

Wanida Mala; wanida.ma@wu.ac.th

Polrat Wilairatana; polrat.wil@mahidol.ac.th

Kwuntida Uthaisar Kotepui; kwuntida.ut@wu.ac.th

Table S1. Search term.

**Databases**	**Search Terms/Search Strategy**	**Date**
MEDLINE	(Malaria OR plasmodium) AND Chikungunya<br>Search results: 441	29 May 2021
Scopus	(Malaria OR plasmodium) AND Chikungunya<br>Search option: Title, abstract, keywords<br>Search results: 876	29 May 2021
ISI Web of Science	(Malaria OR plasmodium) AND Chikungunya	29 May 2021

	Search option: All fields<br><br>Search results: 607	
