# Supplementary material for: Prevalence of Malaria and Chikungunya Co-Infection in Febrile Patients: A Systematic Review and Meta-Analysis
Source: Trop Med Infect Dis. 2021 Jun 30;6(3):119. doi: 10.3390/tropicalmed6030119 (PMC8293423; doi:10.3390/tropicalmed6030119)
Supplement: Supplementary file 1 [file tropicalmed-06-00119-s001.zip › Table S2. Quality of the included studies.pdf]

Prevalence of malaria and Chikungunya co-infection among febrile patients: a systematic review and meta-analysis

Wanida Mala[1], Polrat Wilairatana[2], Kwuntida Uthaisar Kotepui[1], Manas Kotepui[1*]

[1]Medical Technology, School of Allied Health Sciences, Walailak University, Tha Sala, Nakhon Si Thammarat, Thailand

[2]Department of Clinical Tropical Medicine, Faculty of Tropical Medicine, Mahidol University, Bangkok, Thailand

[*]Corresponding author

Manas Kotepui; manas.ko@wu.ac.th, Tel.: +66954392469

Wanida Mala; wanida.ma@wu.ac.th

Polrat Wilairatana; polrat.wil@mahidol.ac.th

Kwuntida Uthaisar Kotepui; kwuntida.ut@wu.ac.th

No.	Authors	Eligibility criteria	Study subjects and the setting	Exposure measured in a valid and reliable way 'gold standard'	A specified diagnosis or definition	Confounding factors	Dealing with confounding factors	Outcomes measured in a valid and reliable way	Appropriate statistical analysis	Scores (8)	Risk of bias (high, moderate, low)
1	Ayorinde et al., 2016	Yes	Yes	No	Yes	No	NA	Yes	Yes	6	Moderate
2	Baba et al., 2013	Yes	Yes	No	Yes	No	NA	Yes	Yes	6	Moderate
3	Bower et al., 2021	Yes	Yes	No	Yes	No	NA	Yes	Yes	6	Moderate
4	Chipwaza et al., 2014	Yes	Yes	No	Yes	No	NA	Yes	Yes	6	Moderate

5	Dariano et al., 2017	Yes	No	No	Yes	No	NA	Yes	Yes	6	Moderate
6	Forero-Peña et al., 2021	Yes	Yes	No	Yes	No	NA	Yes	Yes	6	Moderate
7	Kinimi et al., 2018	Yes	Yes	No	Yes	No	NA	Yes	Yes	6	Moderate
8	Mugabe et al., 2018	Yes	Yes	No	Yes	No	NA	Yes	Yes	6	Moderate
9	Sow et al., 2016	Yes	Yes	Yes	Yes	No	NA	Yes	Yes	7	Low
10	Waggoner et al., 2017	Yes	Yes	Yes	Yes	No	NA	Yes	Yes	7	Low

NA, Not Applicable
